# Larval transcriptomic responses of a stony coral, *Acropora tenuis*, during initial contact with the native symbiont, *Symbiodinium microadriaticum*

**DOI:** 10.1038/s41598-022-06822-3

**Published:** 2022-02-21

**Authors:** Yuki Yoshioka, Hiroshi Yamashita, Go Suzuki, Chuya Shinzato

**Affiliations:** 1 Atmosphere and Ocean Research Institute, The University of Tokyo, Kashiwa, Chiba, Japan; 2 Graduate School of Frontier Sciences, The University of Tokyo, Kashiwa, Chiba, Japan; 3 Fisheries Technology Institute, Japan Fisheries Research and Education Agency, Ishigaki, Okinawa Japan

**Keywords:** Cell biology, Molecular biology

## Abstract

Although numerous dinoflagellate species (Family Symbiodiniaceae) are present in coral reef environments, *Acropora* corals tend to select a single species, *Symbiodinium microadriaticum,* in early life stages, even though this species is rarely found in mature colonies. In order to identify molecular mechanisms involved in initial contact with native symbionts, we analyzed transcriptomic responses of *Acropora tenuis* larvae at 1, 3, 6, 12, and 24 h after their first contact with *S. microadriaticum*, as well as with non-native symbionts, including the non-symbiotic *S. natans* and the occasional symbiont, *S. tridacnidorum*. Some gene expression changes were detected in larvae inoculated with non-native symbionts at 1 h post-inoculation, but those returned to baseline levels afterward. In contrast, when larvae were exposed to native symbionts, we found that the number of differentially expressed genes gradually increased in relation to inoculation time. As a specific response to native symbionts, upregulation of pattern recognition receptor-like and transporter genes, and suppression of cellular function genes related to immunity and apoptosis, were exclusively observed. These findings indicate that coral larvae recognize differences between symbionts, and when the appropriate symbionts infect, they coordinate gene expression to establish stable mutualism.

## Introduction

Symbioses are ubiquitous in nature and are intricately involved in adaptation, ecology, and evolution of most life forms^1, 2^. Cnidarians, such as reef-building corals, are associated with endosymbiotic dinoflagellates of the family Symbiodiniaceae^3, 4^. Coral reefs, structurally dependent upon reef-building corals and their symbionts, are the most biologically diverse shallow-water marine ecosystems^5^. Most coral species (~ 71%) acquire algal symbionts directly from the ocean in each generation^6^. The scleractinian coral genus, *Acropora*, the most common and widespread in the Indo-Pacific^7^, harbors *Symbiodinium* or *Durusdinium* in its early life stages^8, 9^ while mature colonies generally harbor *Cladocopium*^10, 11^. In addition, more than half of *Acropora* recruits (~ 70%) at Ishigaki Island, Okinawa Prefecture, Japan, harbor *Symbiodinium*, even though numerous other genera/species of Symbiodiniaceae, including *Cladocopium*, are common in the water column^9^. Host-symbiont specificity can also be extended to the species level, with *S. microadriaticum* predominating (~ 97%) among the *Symbiodinium* taxa in *Acropora* recruits^12^, indicating that *S. microadriaticum* is a native symbiont in early life stages of *Acropora* in Okinawa.

For recognition of beneficial symbionts or harmful pathogens, pattern recognition receptors (PRRs) on surfaces of host cells and microbe-associated molecular patterns (MAMPs) on surfaces of symbionts are thought to be important^13^. In cnidarians, the PRR-MAMP system is also crucial to establish symbiotic relationships^3^. Cell surfaces of symbiotic dinoflagellates are populated with glycoconjugates, with some glycan motifs similar among species and others unique to each species^14^. Various lectins, which recognize glycans, have been isolated from corals, suggesting that these are involved in recognition of specific symbiotic partners of corals^3, 15–17^. After recognition of symbiotic algae, downstream cellular signaling pathways, such as the innate immune system, were modulated to initiate symbiosis^18^. For example, stimulation of the Toll-like receptor (TLR) signaling pathway affects the stability of symbiosis between the sea anemone, *Exaiptasia diaphana*, and microalgae^19^. In corals, several studies involving bleaching treatments of mature colonies have suggested the importance of immunity and apoptosis for their symbioses^20–24^. However, cellular mechanisms that occur in corals and symbionts during initial contact are still unidentified. Although several studies have examined transcriptomic responses of coral larvae to symbiotic dinoflagellates during initial contact^25–28^, no studies have used their native algal symbionts in early coral life stages.

We recently developed an *Acropora* larval system as a model to study symbiont selection and recognition by host corals^29^. Using this system, we previously documented transcriptomic responses of *A. tenuis* during symbiosis establishment with its native symbiont, *S. microadriaticum* (Smic)^30^. To study molecular responses that occur in coral larvae during initial contact with native symbionts, we analyzed the transcriptome of *A. tenuis* larvae at 1, 3, 6, 12, and 24 h post-inoculation (hpi) with symbionts. In addition, in order to highlight gene expression changes exclusive to native symbionts, we also investigated transcriptomic responses of *A. tenuis* larvae exposed to a closely related, non-symbiotic *Symbiodinium* taxon *S. natans*, (herein Snat), and an occasionally symbiotic *Symbiodinium, S. tridacnidorum* (herein Stri).

## Results

### *Acroporatenuis* larvae express different genes during initial contact with three *Symbiodinium* strains

**Figure 1:**
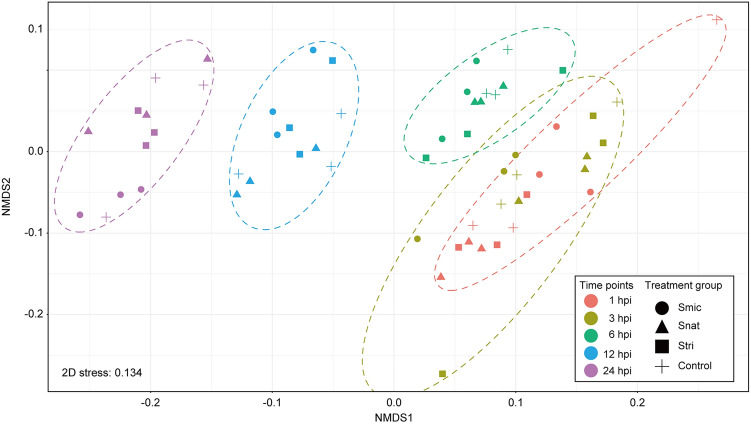
Non-metric multidimensional scaling (NMDS) of RNA-seq samples based on expression levels of *Acropora tenuis* genes. Gene expression levels among all samples were normalized using the trimmed mean of M values method, and then converted to CPM. A total of 5340 genes for which expression levels (TMM-normalized CPM) were larger than 10 in all samples were used with the “metaMDS” of the vegan package^73^. 2D stress was 0.134. Ellipses (dotted line) are drawn around each time point using “geom_mark_ellipse” of the ggplot package^74^. Hpi indicates hours post-inoculation.

Successful infection with each symbiont culture (Smic, Snat, and Stri) was confirmed by fluorescence microscopy in all treatment groups at 24 hpi (Supplementary Fig 1), and proportions of planula larvae with symbiont cells at 24 hpi were about 30% for Smic, 6% for Snat, and 3% for Stri (details are shown in Supplementary Table S1). We performed 3’ mRNA sequencing of *Acropora tenuis* larvae inoculated with Smic, Snat, and Stri and with no *Symbiodinium* exposure (apo-symbiotic) (Supplementary Table S2). At 1, 3, 6, 12, and 24 hpi, gene expression of all *A. tenuis* genes was compared between *Symbiodinium*-exposed and unexposed groups. An average of five million RNA-Seq reads per sample were retained after quality trimming, 65% of which were mapped to *A. tenuis* gene models (n = 22,905, Supplementary Table S2). Non-metric multidimensional scaling (NMDS), based on gene expression levels of 5340 genes for which expression levels (TMM-normalized CPM) were larger than 10 in all samples, showed clear differences in time post-inoculation, but not in treatment groups (Smic-, Snat-, and Stri-inoculated samples and control (apo-symbiotic) samples), indicating that overall transcriptomic states of *A. tenuis* larvae were not significantly affected by symbiont infection and species (Fig. 1). When we compared gene expression levels between Smic-inoculated and control samples, the number of differentially expressed genes (DEGs) gradually increased with inoculation time (three genes at 3 hpi, five at 6 hpi, 106 at 12 hpi, and 392 at 24 hpi; Table 1). In contrast, larvae inoculated with Snat and Stri showed completely different transcriptomic responses (Table 1): 19 genes were differentially expressed in the Snat-inoculated samples and 49 genes in Stri-inoculated samples at 1 hpi (Supplementary Table S3). No gene expression changes were observed at 3 or 12 hpi in the presence of either Snat and Stri, and only one DEG was detected at 6 hpi in Snat- and Stri-inoculated larvae (Supplementary Table S3). Eight DEGs were detected at 24 hpi in Stri-inoculated larvae, but none in Snat-inoculated larvae (Supplementary Table S3).

**Table 1:** Summary of DEG profiles of *Symbiodinium*-inoculated *A. tenuis* larvae at 1, 3, 6, 12, and 24 hpi.

	Number of DEGs				
	1 hpi	3 hpi	6 hpi	12 hpi	24 hpi
Smic-inoculation	0	3	5	106	392
Snat-inoculation	19	0	1	0	0
Stri-inoculation	49	0	1	0	8

Gene expression levels were compared between *Symbiodinium*-exposed and unexposed groups, and genes exhibiting FDR < 0.05 were considered DEGs. Smic, *S. microadriaticum*; Snat, *S. natans*; Stri, *S. tridacnidorum*; hpi, hour post-inoculation.

### Comparison of DEG repertoires between initial contact and symbiosis establishment

**Figure 2:**
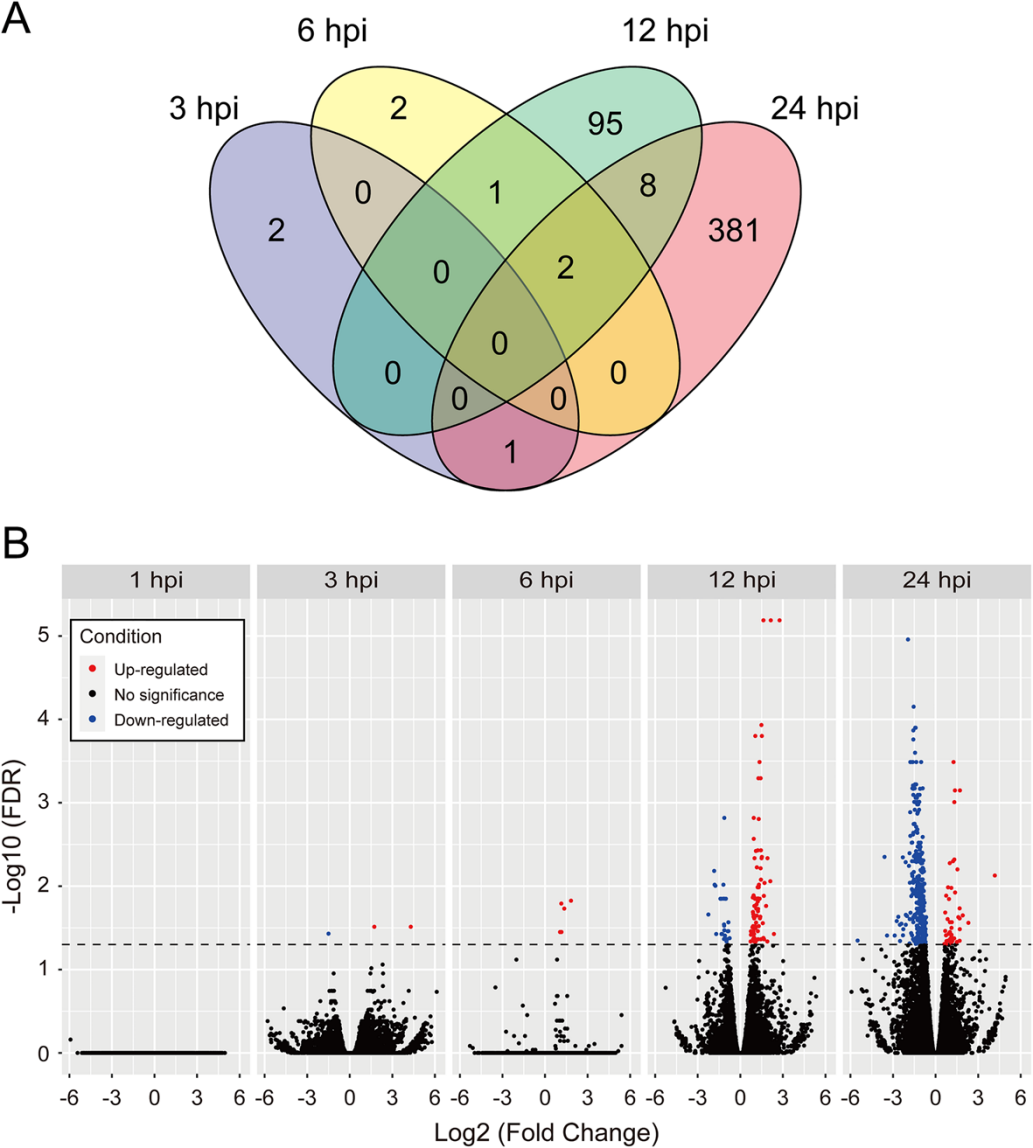
Transcriptomic changes of *Acropora tenuis* larvae during initial contact with native symbionts, *Symbiodinium* *microadriaticum*. (**A**) Comparison of DEG repertoires of Smic-inoculated larvae at 3, 6, 12, and 24 hpi. Raw data are provided in Supplementary Table S3. Hpi indicates hours post-inoculation. (**B**) DEGs that are upregulated or downregulated in Smic-inoculated larvae compared to controls are colored red or blue, respectively. The dotted line indicates FDR = 0.05.

In Smic-inoculated larvae, a limited number of DEGs was shared between time points (3–24 hpi) (Fig. 2A), suggesting that gene expression changes of host corals were drastic during initial contact with native symbionts. Next, we compared DEG repertoires at 24 hpi with a previous study analyzing transcriptomic responses of *A. tenuis* larvae during symbiosis establishment (4, 8, and 12 dpi)^30^. Six DEGs were observed at all time points, 24 hpi, and 4, 8, and 12 dpi (Supplementary Fig. S2), and only 2 upregulated DEGs and 38 downregulated DEGs identified at 24 hpi in this study were also observed at 4 dpi (Supplementary Fig. 2), indicating that DEG repertoires between initial contact and symbiosis establishment are independent, but that the limited array of genes that is constantly differentially expressed in both stages could be important for transition of symbiosis phases.

### *Acroporatenuis* genes that respond to native symbionts during initial contact

We annotated DEGs using BLAST homology searches against the Swiss-Prot database (Supplementary Table S3). Two, five, 80, and 42 genes were upregulated at 3, 6, 12, and 24 hpi, respectively (Fig. 2B). Among those, 0% (0/2 genes), 20% (1/5), 28% (22/80), and 88% (37/42) of DEGs were annotated (Supplementary Table S3). One, 26, and 350 genes were downregulated at 3, 12, and 24 hpi, respectively (Fig. 2B). Of those, 100% (1/1 gene), 69% (18/26), and 70% (246/350) of DEGs were annotated (Supplementary Table S3), indicating that upregulated DEGs involved in initial contact with native symbionts have no homologs that have been annotated yet.

DEGs with Swiss-Prot annotation were used to infer biological processes that occur in Smic-inoculated larvae. To ensure reliability, we focused on categories of UniProt keywords in which more than two annotated genes were detected at each time point. Upregulated DEGs belonging to seven and five categories and downregulated DEGs belonging to one and 28 categories were detected at 12 and 24 hpi, respectively (Table 2). Some categories, such as transport and biological rhythms, were commonly observed among both up- and downregulated DEGs at 24 hpi (Table 2). In *A. tenuis*, seven genes similar to core circadian genes were differentially expressed from 4 to 12 d post-*Symbiodinium* inoculation in the previous study ^30^, and three (*CRY1*: aten_s0034.g64 and aten_s0034.g66; *TIMELESS*: aten_s0021.g100) of them were also differentially expressed in Smic-inoculated larvae at 12 or 24 hpi, or Stri-inoculated samples at 1 hpi (Supplementary Figure S3), indicating that gene expression of core circadian rhythm-regulated genes changed as soon as they were inoculated with *Symbiodinium*. When *A. tenuis* larvae were inoculated with native symbionts, several sugar- and amino acid-transporter genes were specifically upregulated during symbiosis establishment^30^. Nine DEGs possibly involved in transport were upregulated in Smic-inoculated larvae (Supplementary Fig. S4), as were one that contributes to cell volume homeostasis (*SLC12A6*: aten_s0482.g4) and two that may transport sugars or amino acids (*SLC2A12*: aten_s0153.g34, *SLC16A3*: aten_s0261.g16), suggesting that these may be needed to adjust intercellular condition within the symbiosome during initial contact with native symbionts. In addition, in the previous study, two genes, aten_s0153.g34 (*SLC2A12*) and aten_s0482.g4 (*SLC12A6*), were also upregulated at 4 dpi^30^, suggesting their importance during the transition to symbiosis.

**Table 2:** Gene function categories (UniProt Keywords) of differentially expressed genes at 12 and 24 h post-inoculation in Smic-inoculated larvae.

	Annotation term (UniProt Keywords)	Number of genes
Up-regulated at 12 hpi	Transport	6
	Biological rhythms	4
	mRNA processing	4
	Sensory transduction	3
	Transcription	3
	Cell adhesion	2
	Cell cycle	2
	Ubl conjugation pathway	2
Down-regulated at 12 hpi	Transport	6
Up-regulated at 24 hpi	Transcription	4
	Transport	4
	Differentiation	3
	Biological rhythms	2
	Neurogenesis	2
Down-regulated at 24 hpi	Transcription	41
	Cell cycle	40
	Transport	29
	mRNA processing	17
	Cilium biogenesis/degradation	13
	Apoptosis	12
	DNA damage	12
	Differentiation	7
	Endocytosis	6
	Wnt signaling pathway	6
	RNA-mediated gene silencing	5
	Ubl conjugation pathway	5
	rRNA processing	5
	Biological rhythms	4
	Host-virus interaction	4
	Immunity	4
	Inflammatory response	4
	Cell adhesion	3
	DNA recombination	3
	Ribosome biogenesis	3
	Chromosome partition	2
	DNA condensation	2
	DNA replication	2
	Exocytosis	2
	Hearing	2
	Lipid metabolism	2
	Meiosis	2
	Myogenesis	2
	Nonsense-mediated mRNA decay	2
	Protein biosynthesis	2
	Sensory transduction	2

On the other hand, 23 categories of UniProt keywords were exclusively observed among downregulated DEGs at 24 hpi (Table 2). In these categories, transcription and translation (RNA-mediated gene splicing, rRNA processing, ribosome biogenesis, chromosome partition, DNA condensation, nonsense-mediated mRNA decay, and protein biosynthesis), cell proliferation (cell cycle, differentiation, DNA recombination, and myogenesis), bulk transport (endocytosis and exocytosis), and immune response (apoptosis and immunity) were included (Table 2).

### DEGs related to immunity and apoptosis

**Figure 3:**
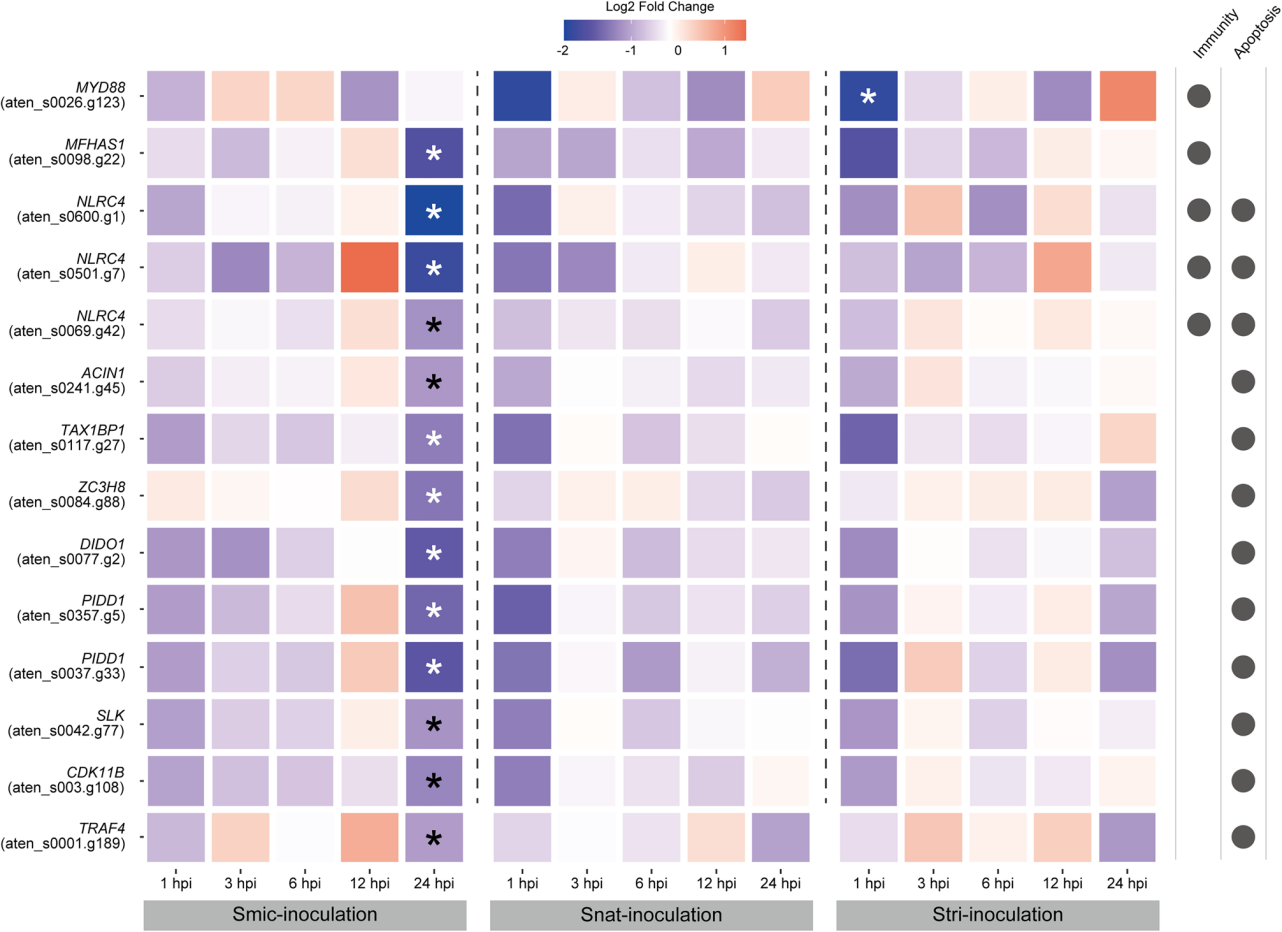
Expression patterns of DEGs related to immunity and apoptosis. DEGs possibly involved in immunity and apoptosis are shown. Possible gene names and gene IDs are shown at the left. Circles on the right indicate functions with which a given gene is associated. “Fold Change” indicates the relative gene expression level compared to controls (apo-symbiotic). *NLRC4*: NLR family CARD domain-containing protein 4. *MFHAS1*: Malignant fibrous histiocytoma-amplified sequence 1. *MYD88*: Myeloid differentiation primary response protein 88. *PIDD1*: p53-induced death domain-containing protein 1. *ACIN1*: Apoptotic chromatin condensation inducer in the nucleus. *TAX1BP1*: Tax1-binding protein 1 homolog. *ZC3H8*: Zinc finger CCCH domain-containing protein 8. *DIDO1*: Death-inducer obliterator 1. *SLK*: STE20-like serine/threonine-protein kinase. *CDK11B*: Cyclin-dependent kinase 11B. *TRAF4*: TNF receptor-associated factor 4.

It is well known that the immune system is modulated during symbiosis establishment in sea anemones ^18, 31, 32^. Four genes (*NLRC4*: aten_s0069.g42, aten_s0501.g7 and aten_s0600.g1; *MFHAS1*: aten_s0098.g22) possibly involved in immunity were significantly downregulated in Smic-inoculated samples at 24 hpi, and one gene (*MYD88*: aten_s0026.g123) was downregulated in Stri-inoculated larvae at 1 hpi (Fig. 3). Apoptosis plays a major role in the host immune response to invading microbes^33^. 12 genes (*NLRC4*: aten_s0069.g42, aten_s0501.g7 and aten_s0600.g1; *ACIN1*: aten_s0241.g45; *TAXBP1*: aten_s0117.g27; *ZC3H8*: aten_s0084.g88; *DIDO1*: aten_s0077.g2; *PIDD1*: aten_s0357.g5 and aten_s0037.g33; *SLK*: aten_s0042.g77; *CDK11B*: aten_s0003.g108; *TRAF4*: aten_s0001.g189) involved in apoptosis were exclusively downregulated in Smic-inoculated larvae at 24 hpi (Fig. 3).

### DEGs related to symbiont recognition and phagocytosis

**Figure 4:**
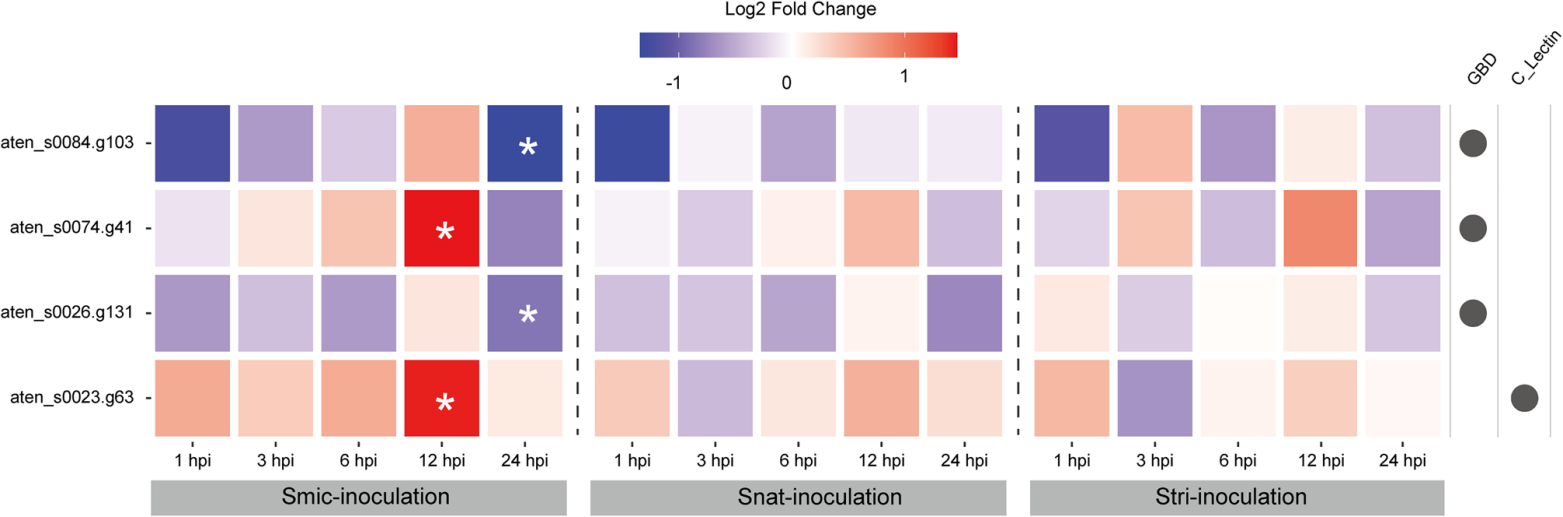
Expression patterns of DEGs related to pattern recognition. DEGs bearing C-type lectin-like superfamily (C_lectin) or galactose-binding domain-like superfamily (GBD) domains are shown. Gene IDs are shown on the left. Circles on the right indicate domains that the gene possesses. “Fold Change” indicates the relative gene expression level compared to controls (apo-symbiotic).

The initial interaction with algal symbionts must involve pattern recognition^3^. Lectin-like genes are important to identify glycans on surfaces of symbionts^15^. We identified 306 genes with lectin-like domains from the *A. tenuis* genome (Supplemental Data S1) and found that four genes (aten_s0084.g103; aten_s0074.g41; aten_s0026.g131; aten_s0023.g63) were exclusively differentially expressed in Smic-inoculated larvae (Fig. 4). Of those, three genes (aten_s0074.g41, aten_s0026.g131 and aten_s0023.g63) were predicted by DeepLoc, a deep learning neural network model, to be localized on the cell membrane. Endocytosis, including phagocytosis, is the main cellular mechanism to acquire symbionts^3^. Among genes involved in this process, one (*STAB2*: aten_s0096.g129) was upregulated, but three genes (*FKBP15*: aten_s0162.g6; *EPS15*: aten_s0079.g89; *MYO6*: aten_s0018.g47) were downregulated in Smic-inoculated larvae (Supplementary Fig. S5). One gene (*LRP4*: aten_s0033.g2) was downregulated in all three samples, but at a different time, and one gene (*APP*: aten_s0027.g17) was exclusively downregulated in Stri-inoculated larvae (Supplementary Fig. S5). On the other hand, two genes (*UNC13B*: aten_s0106.g44l; *MIA3*: aten_s0223.g30) involved in exocytosis were significantly downregulated in Smic-inoculated samples (Supplementary Fig. S5).

### DEGs possibly controlling gene expression for establishment of coral-algal symbiosis

In order to identify genes that may govern coral-algal symbiosis, we focused on signal molecules and transcription factors among DEGs. While eight genes (*HLF*: aten_s0063.g61 and aten_s0156.g13; *TEF*: aten_s0156.g11; *ETS-2*: aten_s0128.g47; *HES4*: aten_s0026.g27; *ZNF271*: aten_s0028.g32; *CIC*: aten_s0075.g3; *GCM2*: aten_s0286.g9) with transcription factor domains were detected as DEGs, no genes with signaling domains were detected (Supplementary Table S4). These genes were not differentially expressed during symbiosis establishment with native symbionts^30^, indicating that they may control the drastic changes in gene expression during initial contact with native symbionts.

## Discussion

A previous study reported that *A. digitifera* larvae immediately changed the expression level of 1,073 genes after exposure (4 hpi) to a non-native symbiont (*Breviolum minutum*), but that no genes were differentially expressed later (at 12 and 24 hpi)^26^. Consistent with the previous study, *A. tenuis* larvae responded to non-native symbionts immediately after inoculation, but expression levels of DEG soon returned to baseline levels (Table 1), suggesting that initial recognition of *Symbiodinium* occurred within 1 h. In contrast, *A. tenuis* larvae gradually responded during initial contact with native symbionts (Table 1). Interestingly, when *A. tenuis* larvae were exposed to *Cladocopium*, a native symbiont of adult corals, the number of DEGs did not increase with infection time^27^, which is different from the results of this study. These differences were probably caused by an infection with symbionts that should not have co-existed in the early life stages in nature. For example, Yuyama et al.^34^ reported that all inoculated *Cladocopium* in *A. tenuis* polyps were abnormal in shape, suggesting that *Cladocopium* may be unsuitable for host corals in early life stages, as the majority of *Acropora* larvae favor *Symbiodinium* or *Durusdinium* in nature^8, 12^.

Symbiotic dinoflagellates possess glycan ligands on their cell surfaces, such as mannose, glucose, and galactose, which are recognized as MAMPs by host corals^15–17^, and lectins that recognize the glycan ligands have been reported from various corals^17, 35–40^. Although continuous gene expression of these lectins should be crucial during initial contact with symbionts, expression of some of them was upregulated when coral larvae were exposed to symbionts^26, 30^. In this study, two genes with lectin-related domains were significantly upregulated only when *A. tenuis* larvae were inoculated with native symbionts (Fig. 4). Interestingly, no genes with lectin-related domains were reportedly differentially expressed when *A. tenuis* was exposed to *Cladocopium*^27^. Considering the specific upregulation of genes with lectin domains to native symbionts in early life stages, these two genes may help to recognize appropriate symbionts in specific life stages.

Dinoflagellates produce diverse photosynthetic products, such as carbohydrates and amino acids^41, 42^, and metabolic exchanges between hosts and symbionts are well known^4^. Upregulation of solute carrier (SLC) transporters, which transport sugars and amino acids, in host corals under daylight^43^ and several days after exposure to native symbionts^30^ have been reported. These SLC transporters are thought to be the major pathway for metabolic exchanges between host corals and symbionts. Although Mohamed et al.^27^ reported upregulation of transporters (*S23A2* and *S26A6*) by 72 hpi with *Cladocopium*, no genes with potential to transport sugars or amino acids were included among DEGs of host corals in that study. In contrast, *SLC2A12*-like gene, which may transport sugars, was upregulated at 24 hpi in this study (Supplementary Fig, S4). Furthermore, this gene was also upregulated at 4 d post-*S. microadriaticum* inoculation^30^, suggesting that nutrient exchange with native symbionts occurs as early as 24 hpi.

Three *NLRC4*-like genes and one *MFHAS1*-like gene were specifically downregulated in Smic-inoculated larvae (Fig. 3). *NLRC4* is a member of the nucleotide oligomerization domain-like receptor (NLR) family^44^, and coral-specific expansion of this group has been reported^45^. NLR can activate several innate immune pathways, including the NFkB and MAPK pathways^44^. *MFHAS1* is a leucine-rich repeat-containing protein and has the potential to modulate the TLR signaling pathway in human macrophages^46^. Although we could not detect downregulation of downstream genes in bulk RNA-seq, these results suggest the occurrence of immune-suppression in Smic-inoculated larvae. On the other hand, an *MYD88*-like gene was significantly downregulated in larvae inoculated with *S. tridacnidorum*, which is an occasional symbiont in early life stages of *Acropora*^12^. *MYD88* is a critical adapter protein downstream of all TLR signaling in mammals^47^, suggesting that immune-suppression may also occur in Stri-inoculated larvae. The importance of immune suppression during symbiosis establishment has been suggested in sea anemones (reviewed in Mansfield and Gilmore^18^), and recently it was experimentally demonstrated in *Aiptasia*^19^, suggesting that immune suppression is conserved and essential for cnidarians during initial contact with their symbionts.

Apoptosis is a highly conserved programmed cell death mechanism in metazoans^48, 49^ and has previously been suggested as a possible pathway in the breakdown of symbiosis under stress in corals^22–24^. Although the possible role of apoptosis in maintenance of a stable symbiotic relationship has not been experimentally addressed, its association during initial contact with symbiotic algae has been suggested, since some apoptosis-related genes were up- and downregulated^25, 50^. Hence, it is thought that apoptosis may contribute to the dynamic equilibrium between host and symbiont cell growth and proliferation^50^. However, another hypothesis has also been proposed by Dunn and Weis^51^. When caspase activity that causes apoptosis was inhibited, larvae of the coral, *Fungia scutaria*, were successfully colonized with a symbiont that is normally unable to colonize; therefore, apoptosis contributes to selection of compatible symbionts after phagocytic uptake^51^. Consistent with this hypothesis, 11 genes involved in apoptosis were exclusively downregulated in larvae inoculated with native symbionts in this study (Fig. 3), indicating that suppression of apoptosis may be conserved among corals as a selection mechanism after phagocytic uptake of symbionts.

In addition to suppression of genes involved in immunity and apoptosis, most DEGs (89.2%) were downregulated at 24 hpi, and functional annotation revealed that many of these encoded transcription and translation, cell proliferation, and immune responses (Table 2), indicating that overall downregulation of cellular functions occurs during initial contact with native symbionts. Although we were unable to detect it in larval transcriptome data until 24 hpi, metabolic suppression of amino acids, sugars, and lipids has been reported in *A. tenuis* larvae at 4–12 dpi^30^; thus, suppression of genes involved in transcription and translation may be related to metabolic suppression.

**Figure 5:**
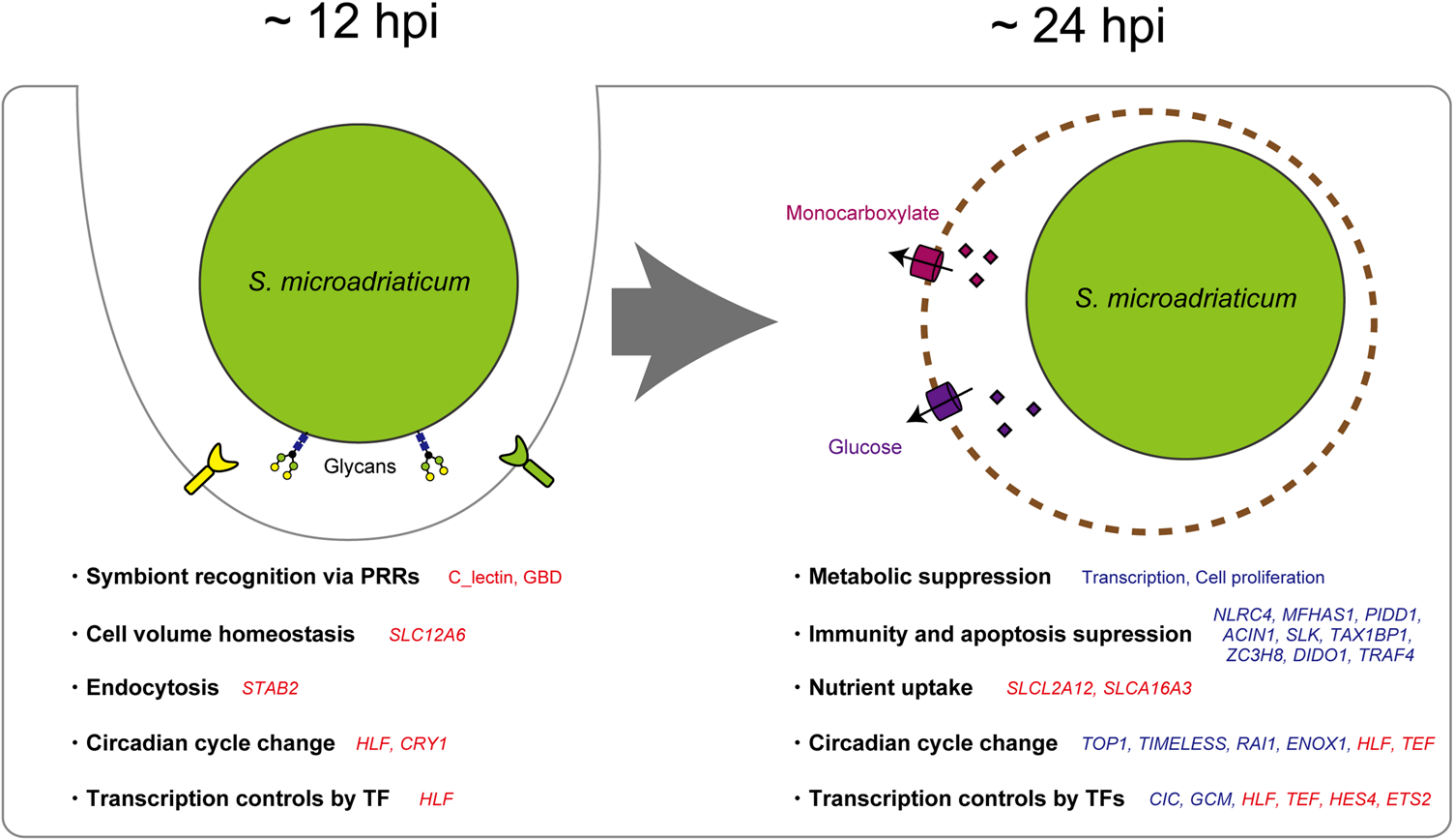
Schematic time series summary of possible intercellular events occurring in *Acropora tenuis* larvae during initial contact with native symbionts. Sentences with a dot indicate possible cellular events, and genes associated with them are shown nearby. A brown dotted line indicates a symbiosome (the organelle in which a symbiont resides). Red or blue text indicates significantly (FDR < 0.05) up- or down-regulated genes, respectively. PRRs indicate pattern recognition receptors. TF indicates transcription factor.

*Symbiodinium* is one of the dominant algal symbionts in early life stages of *Acropora* corals at Ishigaki Island, Okinawa Prefecture, Japan (until they are at least 14 d old) ^12^ and the southern Great Barrier Reef, Australia (until they are at least 83 d old)^52^. One reason for this may be that *Symbiodinium* are highly infectious to corals at this stage^8, 52, 53^. However, another possible reason for this may be that *Symbiodinium* tolerates higher solar irradiance and thermal stress^54–57^. Despite these advantages, adult colonies of *Acropora* in those locations are mainly associated with *Cladocopium*^10, 11, 52^. Perhaps this is because *Symbiodinium* has a lower carbon fixation rate than *Cladocopium*, which is required to form calcium carbonate skeletons^58^. The genus *Symbiodinium* includes species with characteristics ranging from symbiotic to opportunistic or free living^59^. Symbiosis between *Acropora* and *Symbiodinium* differs even among closely related species^29^. Smic (AJIS2-C2) isolated from an *Acropora* recruit is taken up by *A. tenuis* planula larvae more than other *Symbiodinium*^29^. Our transcriptomic data suggest that Smic modulates the immune system of host corals and exchanges metabolites with the host within 24 h (Fig. 5), indicating that highly infectious Smic is a suitable symbiotic partner for *Acropora* in early life stages.

In summary, our data show clear transcriptomic differences in coral larvae in response to native and non-native symbionts, indicating that *A. tenuis* larvae recognize different *Symbiodinium* strains within 1 hpi. When *A. tenuis* larvae contact native symbionts, symbiont recognition, circadian cycle changes, cell volume homeostasis, and endocytic uptake occur within 12 hpi (Fig. 5), and then metabolic suppression, immune and apoptosis suppression, circadian cycle changes, and nutrient uptake are induced by 24 hpi (Fig. 5). This study highlights not only the importance of immune-response suppression and apoptosis suppression during initial contact with native symbionts, but also the relevance of cellular mechanisms, such as circadian cycle changes and nutrient uptake, during the period from initial contact to symbiosis establishment. Although RNA-seq techniques have become more feasible than in the previous decade, it is still difficult to capture minute gene expression changes with bulk RNA-seq, because only a small percentage of the volume of a coral larva contains cells with algae. Tissue-specific RNA-seq^19, 60^, single cell RNA-seq^61^, or coral cell lines^62^ may reveal more comprehensive molecular responses of coral symbioses in the future.

## Methods

### Preparation of *Acroporatenuis* planula larvae and *Symbiodinium* culture strains

Colonies of *A. tenuis* were collected in Sekisei Lagoon, Okinawa, Japan, in May 2018, and were maintained in aquaria at the Yaeyama Station, Fisheries Technology Institute, until spawning. Permits for coral collection were kindly provided by the Okinawa Prefectural Government for research use (Permits 29-74). After fertilization, we washed the embryos with seawater passed through a 0.2-µm filter (FSW) to remove unwanted contaminants. Embryos were maintained at a concentration of ~ 2 individuals per mL of FSW in plastic bottles at 23.6 ± 0.7 °C. FSW was changed once a day until planula larvae reached 6 d post-fertilization.

We used three *Symbiodinium* culture strains, AJIS2-C2 (*S. microadriaticum*), CS-161 (*S. tridacnidorum*) and ISS-C2-Sy (*S. natans*) in this study. The culture strain AJIS-C2 was originally isolated from *Acropora* recruits^63^. Culture strain CS-161, which has occasionally been detected in wild corals, was purchased from the Australian National Algae Culture Collection, Australia. Culture strain ISS-C2-Sy was originally isolated from coral reef sand at Ishigaki Island^63^.

### Inoculation experiments

*A. tenuis* larvae were divided into four treatment groups, with three replicates per treatment. Inoculation experiments using the three culture strains were conducted as in Yamashita et al.^29^. In each replicate, ~ 1500 *A. tenuis* planula larvae at 6 d post-fertilization were placed in 2-L bottles containing 1500 mL of FSW. Smic, Snat and Stri strains at 50 cells/mL were added to the first to third group of larvae, respectively. The remaining group was used as control (apo-symbiotic). All bottles were kept at 23.6 ± 0.7 °C. At 24 hpi, 10 randomly selected larvae from each bottle were used for observation of the algae under a fluorescence microscope (BX50; Olympus; 400–410 nm excitation) to determine whether they were infected with *Symbiodinium*.

### RNA extraction, sequencing, and transcriptomic analyses

At 1, 3, 6, 12, and 24 hpi, ~ 300 planula larvae from each bottle were collected and stored at − 80 °C until use. Coral larvae were homogenized with zirconia beads (ZB-20) in TRIzol reagent (Thermo Fisher Scientific) using a beads beater (TOMY Micro Smash MS-100) at 3000 rpm for 10 s. Total RNA was extracted from each larva using TRIzol reagent according to the manufacturer’s protocol. A Collibri 3’ mRNA Library Prep Kit for Illumina (Thermo Fisher Scientific) was used for sequencing library preparations. Sequencing adaptors were attached by PCR amplification with 16 cycles of annealing according to the manufacturer’s protocol. Each library was sequenced on a NovaSeq 6000 (Illumina) with 50-bp, single-end reads. Low-quality reads (quality score < 20 and length < 20-bp) and Illumina sequence adaptors were trimmed with CUTADAPT v1.16^64^. Then cleaned reads were mapped to the *A. tenuis* gene model (mRNA) using BWA v0.7.17^65^ with default settings. Transcript abundances in each sample were quantified using SALMON v1.0.0^66^. Mapping counts were normalized by the trimmed mean of M values (TMM) method, and then converted to counts per million (CPM) using EdgeR v3.32.1^67^ in R v4.0.3^68^. Gene expression levels (numbers of mapped reads) in treatment groups were compared with control samples (apo-symbiotic) to identify DEGs. Obtained *p*-values were adjusted using the Benjamini–Hochberg method in EdgeR. When the gene expression level was significantly different (False discovery rate < 0.05) than control samples, genes were considered DEGs. We downloaded gene models of *A. tenuis*^69^ from the genome browser of the OIST Marine Genomics Unit (https://marinegenomics.oist.jp). Gene models were annotated with BLASTP^70^ and InterProScan^71^ against the Swiss-Prot database and Pfam database as described in Yoshioka et al.^30^. Putative transposable elements in gene models were identified with Pfam keywords (“Transposase”, “Integrase”, and “Reverse transcriptase”) and were excluded from analyses in this study. Subcellular localization of lectin-like genes was predicted using the DeepLoc-1.0 online server^72^.

## Supplementary Information

Supplementary Information 1.

Supplementary Information 2.

Supplementary Information 3.

Supplementary Information 4.

Supplementary Information 5.

## Data Availability

Raw RNA sequencing data were deposited in the DDBJ/EMBL/GenBank databases under accession number DRA013077 (BioProject ID: PRJDB8332). A genome browser for *A. tenuis* is available from the Marine Genomics Unit web site (https://marinegenomics.oist.jp/). Results of statistical analyses for identify DEGs were provided in Supplementary Data S2. Normalized expression data (TMM-normalized CPM) was provided in Supplementary Data S3.
