# Supplementary material for: Larval transcriptomic responses of a stony coral, Acropora tenuis, during initial contact with the native symbiont, Symbiodinium microadriaticum
Source: Sci Rep. 2022 Feb 21;12:2854. doi: 10.1038/s41598-022-06822-3 (PMC8861010; doi:10.1038/s41598-022-06822-3)
Supplement: Supplementary file 4 — Supplementary Information 4. [file 41598_2022_6822_MOESM4_ESM.docx]

Supplementary information for
“**Larval transcriptomic responses of a stony coral, *Acropora tenuis*, during initial contact with the native symbiont, *Symbiodinium microadriaticum***”

Yuki Yoshioka ^1, 2^, Hiroshi Yamashita ^3^, Go Suzuki ^3^ and Chuya Shinzato ^1*^

^1^ Atmosphere and Ocean Research Institute, The University of Tokyo, Kashiwa, Chiba, Japan

^2^ Graduate School of Frontier Sciences, The University of Tokyo, Kashiwa, Chiba, Japan

^3^ Fisheries Technology Institute, Japan Fisheries Research and Education Agency, Ishigaki, Okinawa, Japan

^*^Corresponding authors:

Chuya Shinzato (c.shinzato@aori.u-tokyo.ac.jp)

**Supplementary Table S1. Number of planula larvae with at least one symbiont cells.**

|  | Smic-inoculation | | | Snat-inoculation | | | Stri-inoculation | | |
| --- | --- | --- | --- | --- | --- | --- | --- | --- | --- |
|  | Bottle 1 | Bottle 2 | Bottle 3 | Bottle 1 | Bottle 2 | Bottle 3 | Bottle 1 | Bottle 2 | Bottle 3 |
| No. of infected larvae per 10 planula larvae observed | 3 | 2 | 4 | 0 | 1 | 1 | 1 | 0 | 0 |

**Supplementary Table S2. Summary of numbers of mapped reads to *A. tenuis* gene models.**

^*1^: hpi indicates hour post-inoculation.

| Samples ^*1^ | No. of reads after quality trimming | No. of mapped reads |
| --- | --- | --- |
| contorl 1 at 1 hpi | 5,705,983 | 2,296,130 |
| control 2 at 1 hpi | 5,143,173 | 1,419,187 |
| control 3 at 1 hpi | 5,268,862 | 2,210,749 |
| control 1 at 3 hpi | 3,690,170 | 3,370,026 |
| control 2 at 3 hpi | 2,621,040 | 2,668,196 |
| control 3 at 3 hpi | 3,823,458 | 4,002,757 |
| control 1 at 6 hpi | 2,115,241 | 3,384,543 |
| control 2 at 6 hpi | 5,053,028 | 3,887,936 |
| control 3 at 6 hpi | 5,192,290 | 2,255,906 |
| control 1 at 12 hpi | 5,980,499 | 3,836,008 |
| control 2 at 12h | 4,968,612 | 3,631,682 |
| control 3 at 12h | 6,695,452 | 3,526,527 |
| control 1 at 24h | 5,193,541 | 1,255,255 |
| control 2 at 24h | 6,058,130 | 3,289,758 |
| control 3 at 24h | 3,680,061 | 3,658,801 |
| Smic 1 at 1 hpi | 3,585,960 | 2,125,531 |
| Smic 2 at 1 hpi | 4,410,433 | 2,664,577 |
| Smic 3 at 1 hpi | 3,804,153 | 2,290,027 |
| Smic 1 at 3 hpi | 2,756,219 | 1,573,715 |
| Smic 2 at 3 hpi | 8,847,247 | 5,250,556 |
| Smic 3 at 3 hpi | 7,970,525 | 4,278,826 |
| Smic 1 at 6 hpi | 5,716,404 | 3,627,541 |
| Smic 2 at 6 hpi | 7,919,985 | 4,846,590 |
| Smic 3 at 6 hpi | 5,609,998 | 3,510,024 |
| Smic 1 at 12 hpi | 5,681,112 | 3,783,347 |
| Smic 2 at 12 hpi | 1,758,993 | 1,024,542 |
| Smic 3 at 12 hpi | 8,609,147 | 6,018,124 |
| Smic 1 at 24 hpi | 4,429,262 | 3,039,708 |
| Smic 2 at 24 hpi | 5,166,413 | 3,520,161 |
| Smic 3 at 24 hpi | 4,639,482 | 3,153,920 |
| Snat 1 at 1 hpi | 4,165,988 | 2,682,006 |
| Snat 2 at 1 hpi | 3,963,959 | 2,757,234 |
| Snat 3 at 1 hpi | 1,821,971 | 1,186,389 |
| Snat 1 at 3 hpi | 6,532,594 | 3,570,644 |
| Snat 2 at 3 hpi | 6,475,958 | 3,787,330 |
| Snat 3 at 3 hpi | 5,794,120 | 3,478,356 |
| Snat 1 at 6 hpi | 5,703,346 | 3,583,270 |
| Snat 2 at 6 hpi | 10,288,719 | 6,680,691 |
| Snat 3 at 6 hpi | 5,251,945 | 3,273,010 |
| Snat 1 at 12 hpi | 5,292,794 | 3,474,913 |
| Snat 2 at 12 hpi | 6,012,810 | 4,323,165 |
| Snat 3 at 12 hpi | 5,685,154 | 4,038,608 |
| Snat 1 at 24 hpi | 5,150,341 | 3,302,348 |
| Snat 2 at 24 hpi | 6,492,303 | 4,043,905 |
| Snat 3 at 24 hpi | 2,453,083 | 1,522,499 |
| Stri 1 at 1 hpi | 5,473,598 | 3,556,007 |
| Stri 2 at 1 hpi | 4,662,541 | 3,013,836 |
| Stri 3 at 1 hpi | 4,932,684 | 3,291,767 |
| Stri 1 at 3 hpi | 7,419,793 | 4,153,421 |
| Stri 2 at 3 hpi | 6,566,772 | 3,746,971 |
| Stri 3 at 3 hpi | 486,073 | 215,084 |
| Stri 1 at 6 hpi | 5,289,580 | 3,419,574 |
| Stri 2 at 6 hpi | 5,319,678 | 3,437,006 |
| Stri 3 at 6 hpi | 4,937,276 | 3,275,967 |
| Stri 1 at 12 hpi | 6,138,445 | 4,283,990 |
| Stri 2 at 12 hpi | 5,215,240 | 3,585,984 |
| Stri 3 at 12 hpi | 7,267,536 | 4,261,939 |
| Stri 1 at 24 hpi | 1,630,191 | 976,355 |
| Stri 2 at 24 hpi | 853,348 | 528,512 |
| Stri 3 at 24 hpi | 1,233,947 | 741,547 |

**Supplementary Table S3. Summary of DEGs detected in each treatment group.**

Attached separately (SupTableS3.xlsx) due to file size.

**Supplementary Table S4. Summary of DEGs with transcription factor domains.**

^*1^: hpi indicates hour post-inoculation.

| Gene ID | Pfam domain | Swiss-Prot annotation | Differentially expressed at*^1^ | Log2 Fold Change |
| --- | --- | --- | --- | --- |
| aten_s0156.g13 | Basic region leucine zipper (PF07716) | Hepatic leukemia factor | 12-hpi | 2.763 |
| aten_s0063.g61 | Basic region leucine zipper (PF07716) | Hepatic leukemia factor | 12-hpi 24-hpi | 0.979 1.000 |
| aten_s0156.g11 | Basic region leucine zipper (PF07716) | Thyrotroph embryonic factor | 24-hpi | 0.874 |
| aten_s0128.g47 | Ets-domain (PF00178) | Protein C-ets-2 | 24-hpi | 0.753 |
| aten_s0026.g27 | Helix-loop-helix DNA-binding domain (PF00010) | Transcription factor HES-4 | 24-hpi | 1.119 |
| aten_s0028.g32 | Zinc finger, C2H2 type (PF00096) C2H2-type zinc finger (PF13894) | NA | 24-hpi | -1.375 |
| aten_s0075.g3 | HMG (high mobility group) box (PF00505) | Protein capicua homolog | 24-hpi | -1.539 |
| aten_s0286.g9 | GCM motif protein (PF03615) | Transcription factor glial cells missing | 24-hpi | -1.01 |

**
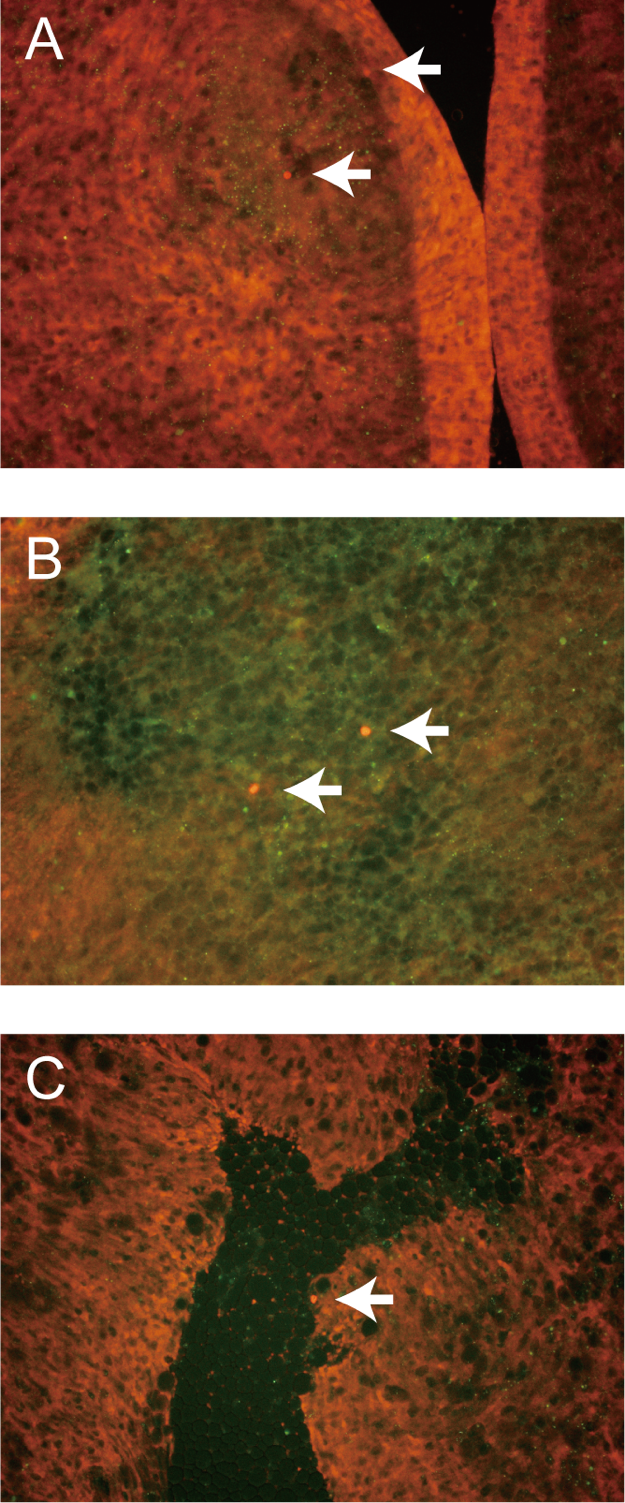
**

**Supplementary Figure S1. High magnification photos of *A. tenuis* larvae containing inoculated *Symbiodinium* strains.**

(A) *A. tenuis* larvae inoculated with *S. microadriaticum*.

(B) *A. tenuis* larvae inoculated with *S. natans*.

(C) *A. tenuis* larvae inoculated with *S. tridacnidorum*.

White arrows indicate a *Symbiodinium* cell in coral larvae. Orange and cyan-green fluorescence shows fluorescent proteins from *A. tenuis*. Red fluorescence shows chlorophyll in algal symbionts.

**
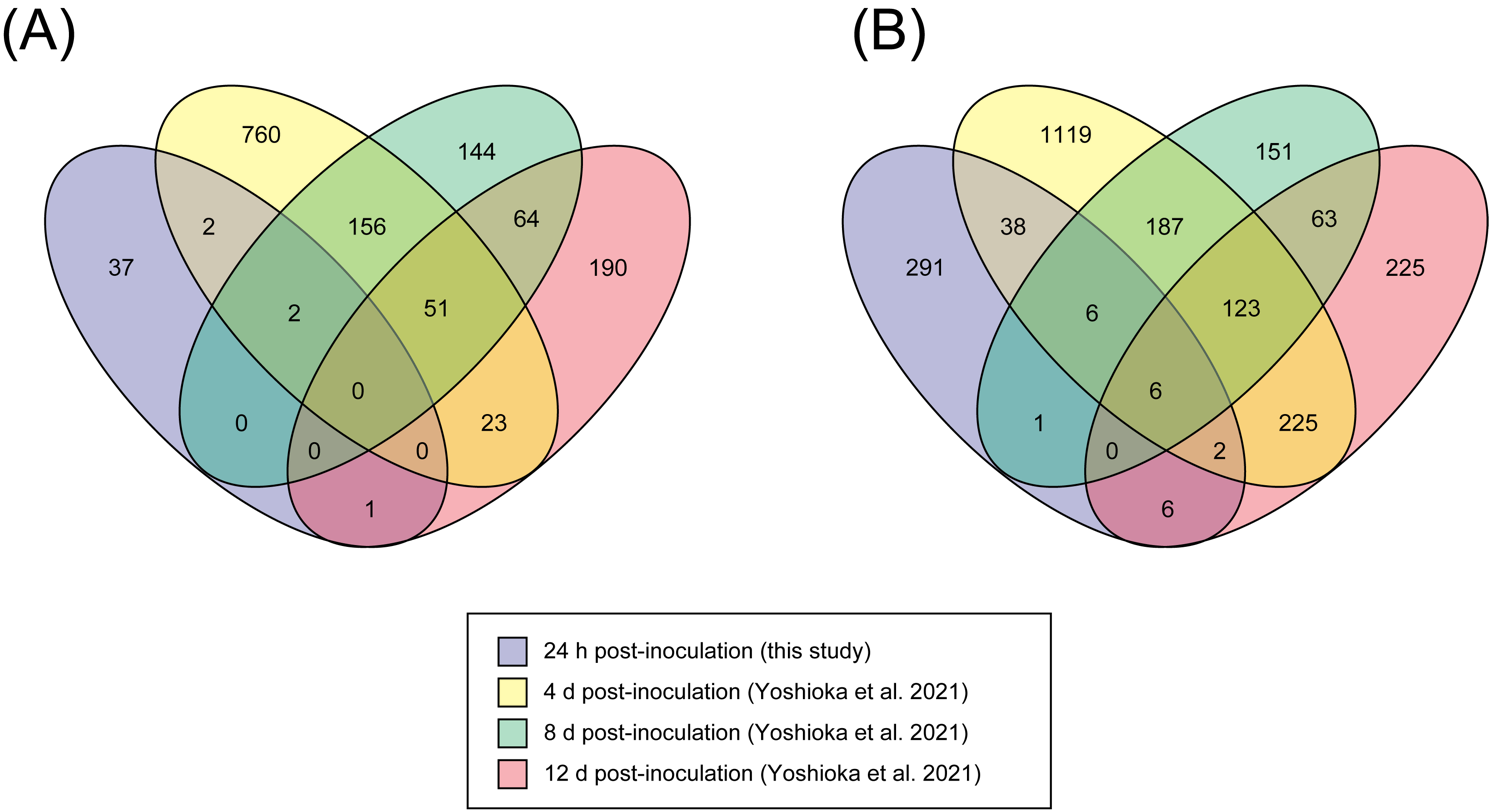
**

**Supplementary Figure S2. Comparison of DEG repertoires 24-hpi (this study), 4-, 8-, and 12-dpi (Yoshioka et al. 2021).**

(A) Comparison of DEG repertoires that were upregulated in all samples compared with their respective controls.

(B) Comparison of DEG repertoires that were downregulated in all samples compared with their respective controls.

Genes exhibiting FDR < 0.05 were considered as DEGs. DEGs at 4-, 8-, 12-dpi were retrieved from Yoshioka et al ^1^.

**
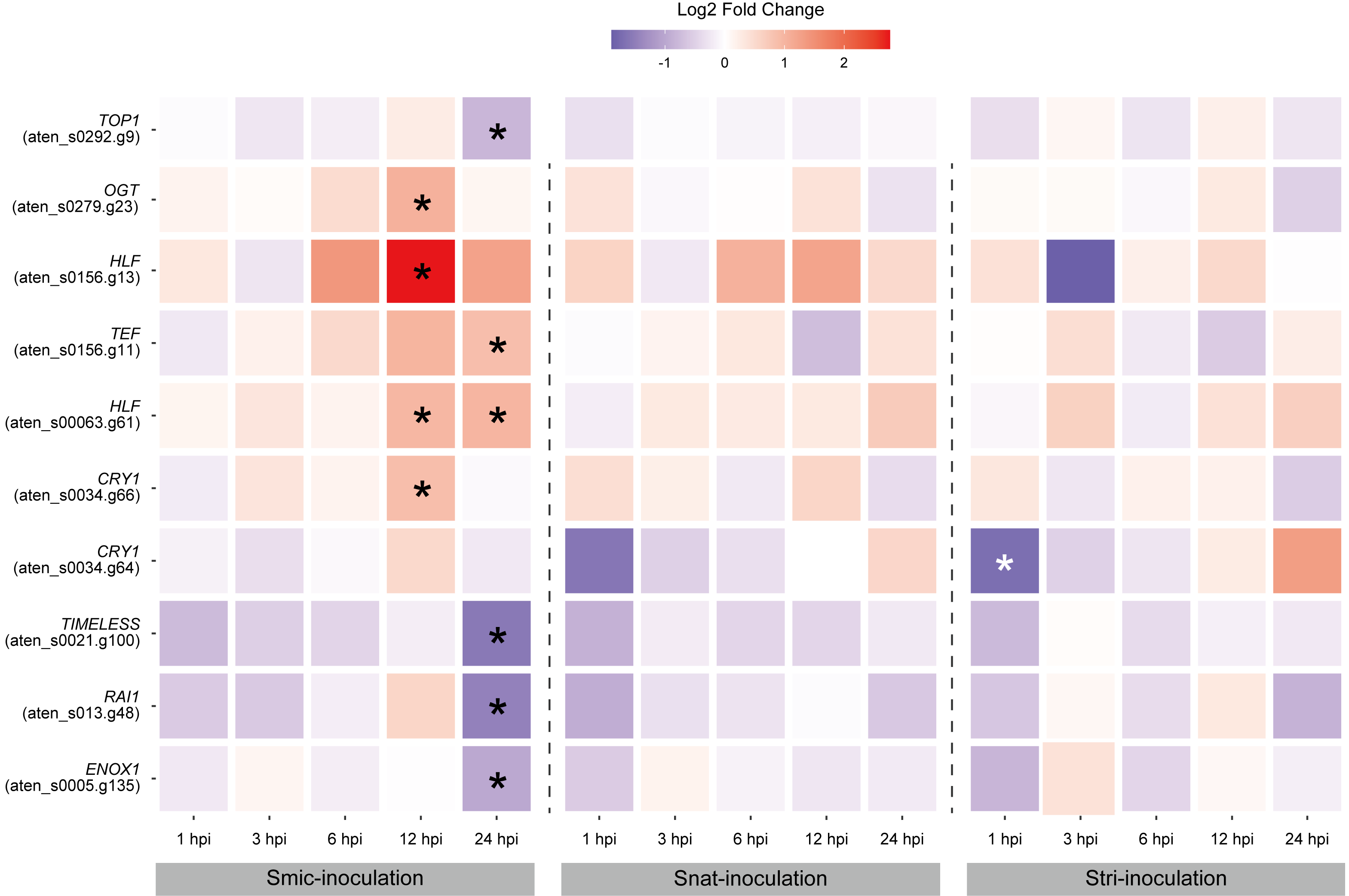
**

**Supplementary Figure S3. Expression patterns of DEGs related to circadian rhythms.**

Possible gene names and gene IDs are shown at the left. “Fold Change” indicates relative gene expression levels compared with controls (apo-symbiotic). Asterisks indicate significant differences (FDR < 0.05) compared with controls.

*TOP1*: DNA topoisomerase 1. *OGT*: UDP-N-acetylglucosamine-peptide N-acetylglucosaminyltransferase 110 kDa subunit. *HLF*: Hepatic leukemia factor. *TEF*: Thyrotroph embryonic factor. *CRY1*: Cryptochrome-1. *TIMELESS*: Protein timeless homolog. *RAI1*: Retinoic acid-induced protein 1. *ENOX1*: Ecto-NOX disulfide-thiol exchanger 1.

**
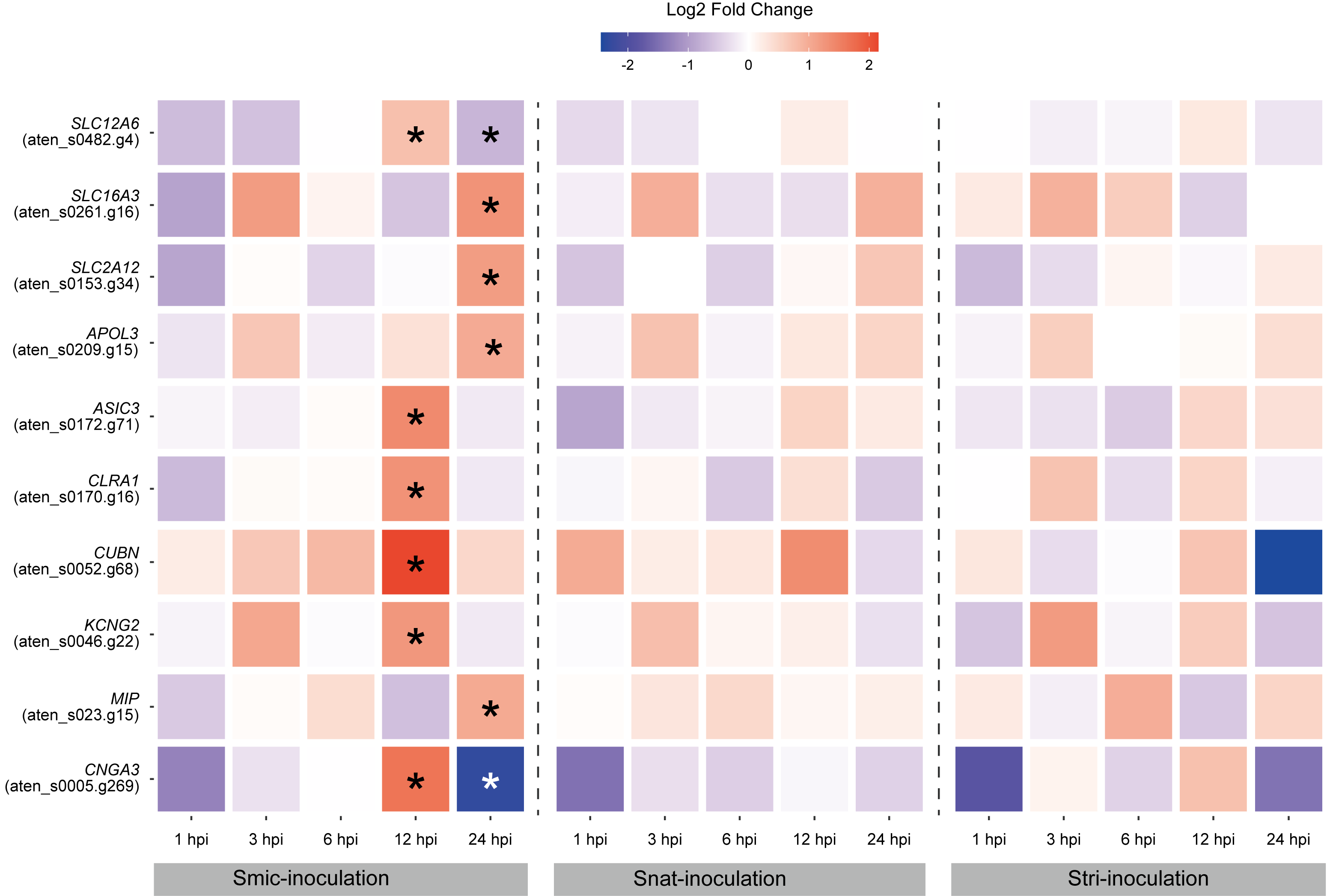
**

**Supplementary Figure S4. Expression patterns of upregulated DEGs related to transport**.

Possible gene names and gene IDs are shown at the left. “Fold Change” indicates relative gene expression levels compared withs controls (apo-symbiotic). Asterisks indicate significant differences (FDR < 0.05) compared with controls.

*SLC12A6*: Solute carrier family 12 member 6. *SLC16A3*: Monocarboxylate transporter 3. *APOL3*: Apolipoprotein L3. *ASIC3*: Acid-sensing ion channel 3. *GLRA1*: Glycine receptor subunit alpha 1. *SLC2A12*: Solute carrier family 2, facilitated glucose transporter member 12. *CUBN*: Cubilin. *KCNG2*: Potassium voltage-gated channel subfamily G member 2. *MIP*: Lens fiber major intrinsic protein. *CNGA3*: Cyclic nucleotide-gated cation channel alpha-3.

**
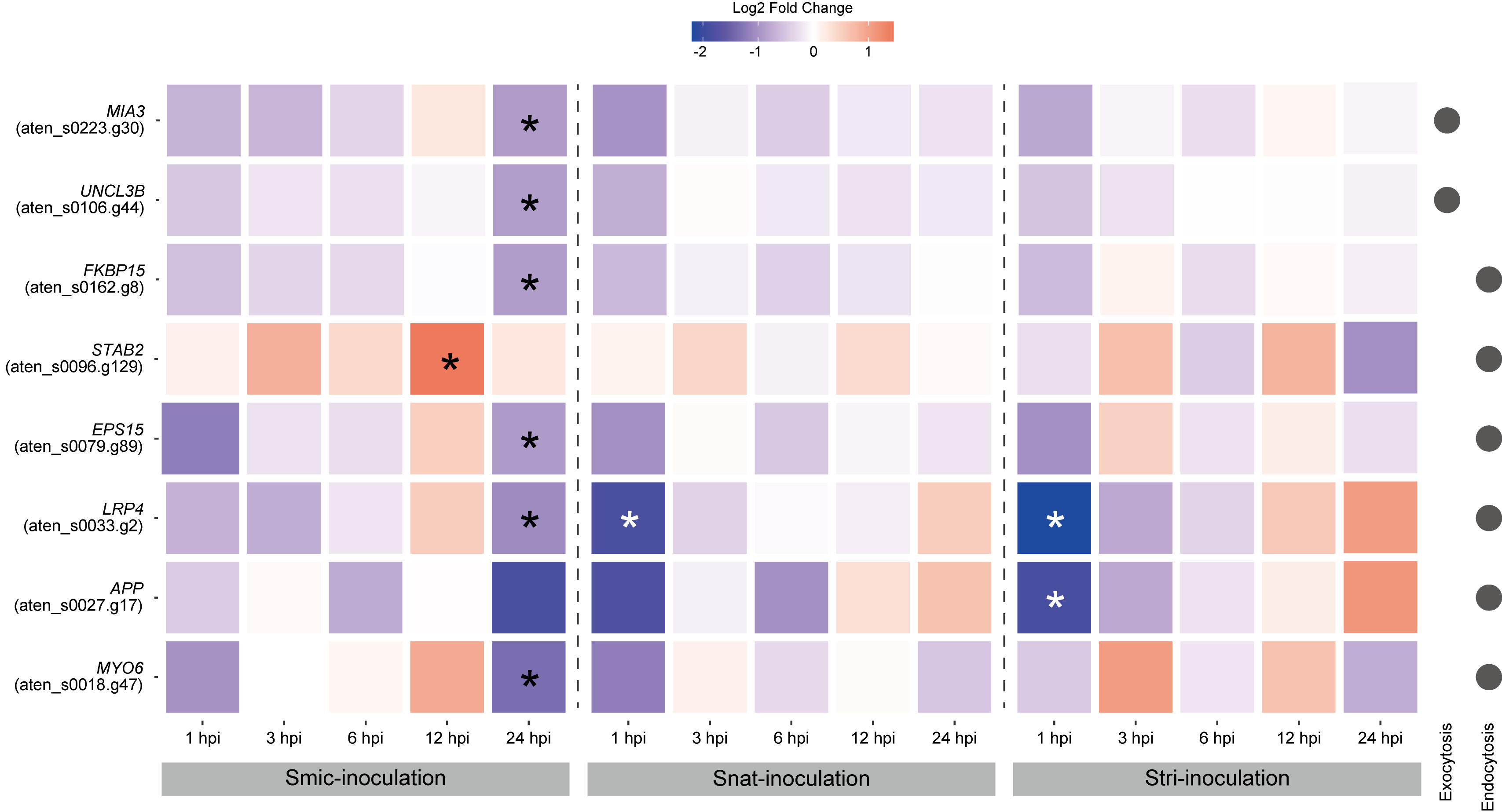
**

**Supplementary Figure S5. Expression patterns of DEGs related to endocytosis and exocytosis.**

Possible gene names and gene IDs are shown in at the left. “Fold Change” indicates relative gene expression levels compared with controls (apo-symbiotic). Asterisks indicate significant differences (FDR < 0.05) compared with controls.

*MIA3*: Melanoma inhibitory activity protein 3. *FKBP15*: FK506-binding protein 15. *UNC13B*: Protein unc-13 homolog B. *STAB2*: Stabilin-2. *EPS15*: Epidermal growth factor receptor substrate 15. *LRP4*: Low-density lipoprotein receptor-related protein 4. *APP*: Amyloid beta A4 protein. *MYO6*: Unconventional myosin-VI.

**References**

1. Yoshioka, Y. *et al*. Whole-Genome Transcriptome Analyses of Native Symbionts Reveal Host Coral Genomic Novelties for Establishing Coral–Algae Symbioses. *Genome biology and evolution* **13**, evaa240 (2021).stylefix
